# Analysis of clinical and physical dosimetric factors that determine the outcome of severe acute radiation pneumonitis in lung cancer patients

**DOI:** 10.1186/s13014-023-02304-6

**Published:** 2023-08-29

**Authors:** Jing Zhao, Chenying Ma, Guanghui Gan, Xiaoting Xu, Juying Zhou

**Affiliations:** Department of Radiation Oncology, First Affiliated Hospital of Soochow University, Suzhou, 215000 China

**Keywords:** Lung cancer, Intensity-modulated radiotherapy, Radiation pneumonitis, SARP

## Abstract

**Objective:**

We conducted a retrospective statistical analysis of clinical and physical dosimetric factors of lung cancer patients who had previously undergone lung and/or mediastinal radiotherapy and died of or survived severe acute radiation pneumonitis (SARP). Our study was the first to reveal the heterogeneity in clinical factors, physical dosimetric factors, and SARP onset time that determined the clinical outcomes of lung cancer patients who developed SARP.

**Materials and methods:**

The clinical characteristics, physical dosimetry factors, and SARP onset time of deceased and surviving patients were retrospectively analyzed. SPSS 20.0 was used for data analysis. Student’s t-test was used for intergroup comparison, and a Mann–Whitney *U* test was used for data with skewed distribution. Qualitative data were represented using frequencies (%), and Fisher’s exact test or *χ*^2^ test was used for intergroup comparison of nonparametric data. Binary logistic analysis was used for univariate and multivariate analyses. Differences with a P < 0.05 were considered statistically significant.

**Results:**

Univariate analysis revealed that the potential predictors of SARP death were as follows: ipsilateral lung V5 and V30, contralateral lung V5, V10, and V30, total lung V5, V10, and V30, mean lung dose, mean heart dose, and maximum spinal cord dose. Multivariate analysis showed that ipsilateral lung V5 and total lung V5 were predictors that determined the final outcome of SARP patients. In addition, we analyzed the time from the completion of radiotherapy to SARP onset, and found significant difference between the two groups.

**Conclusions:**

There was no decisive correlation between clinical characteristics and SARP outcome (i.e., death or survival) in lung radiotherapy patients. Ipsilateral lung V5 and total lung V5 were independent predictors of death in SARP patients.

## Introduction

Lung cancer is still one of the leading malignancies that threaten human health and life globally. The incidence and mortality rates of lung cancer are ranked first among all malignancies in males and second in females [1–3]. Radiotherapy (RT) is one of the mainstay treatments for lung cancer patients and can provide postoperative adjuvant therapy in patients with resectable lung cancer and treatment support in patients with unresectable lung cancer [4, 5]. Radiation pneumonitis (RP) is a common inflammatory response seen within 6 months after chest and mediastinal radiotherapy and is caused by the radiation damage to normal lung tissues in the radiation volume [4, 5]. RP can cause chronic lung insufficiency if not promptly or properly treated. This can affect the quality of life of patients and even cause poor outcomes and death [6, 7].

The Radiation Therapy Oncology Group (RTOG) classified RP into grades 0 to 5 based on the clinical symptoms, lung radiologic presentation, and corticosteroid treatment results. After discontinuing radiotherapy, some patients develop severe acute RP (SARP), i.e., grade 3 or above [8]. Most previous studies on RP have focused on clinical factors, such as smoking, lung function, presence of comorbid chronic obstructive pulmonary disease (COPD), and pulmonary fibrosis, and physical dosimetric factors, such as the percentage of ipsilateral lung volume receiving 5 Gy, 20 Gy, and 30 Gy (V5, V20, and V30), mean lung dose (MLD), and heart dose [9, 10]. In addition, relevant prediction models were constructed to guide clinical treatment so as to decrease the severity of RP. Even so, SARP still occurs after lung cancer radiotherapy and its incidence is 3–6%. However, clinical outcomes of these patients are different in clinical practice, i.e., death or survival [11–13].

Clinical and dosimetric factors that determine the clinical outcomes of lung cancer patients with SARP are still unknown. There have been no related studies that compared the radiation dosimetric parameters between patients with different outcomes and evaluated their role in treatment-related toxicity. Therefore, we focused on the final outcome of SARP patients and attempted to determine the heterogeneity in clinical factors and radiotherapy dosimetric parameters in lung cancer patients with grade 3 and above RP who had different outcomes. This approach could provide a theoretical research foundation for further optimization of lung radiotherapy plan and decreasing the incidence of fatal RP in lung cancer patients in clinical practice.

## Materials and methods

### Patient enrollment

Data were collected from 66 lung cancer patients who had undergone lung and/or mediastinal radiotherapy and developed SARP from 2013 to 2019 in our center. There were 31 deaths and 35 survivors. The inclusion criteria were as follows: (1) clinicopathologically confirmed lung cancer; (2) lung and/or mediastinum received 40–65 Gy radiotherapy; (3) complete and available clinicopathological and physical dosimetric parameters; (4) satisfactory general condition of the patient during radiotherapy. The exclusion criteria were as follows: (1) severe cardiac, hepatic, or renal impairment; (2) discontinuation of treatment due to severe complications during and after radiotherapy (e.g., cardiac, pulmonary, hepatic, or renal impairment, severe infection, severe myelosuppression, or hemoptysis); (3) previous history of chest radiotherapy; (4) lack of complete and periodic radiologic diagnosis when RP occurred; (5) acute infection or autoimmune disease. This study was approved by the Ethics Committee of the hospital. Every subject was given an informed consent form for participating in this study, and approval was granted for data collection.

### Collection and definition of clinical information and biological parameters

Clinical information, including the general condition, personal history, tumor stage, pathological and radiologic information, and treatment status of the patient, was obtained from the doctor’s workstation (Haitai) and the imaging system (PECS) of this center. Clinical data included patient’s age, gender, ECOG (Eastern Cooperative Oncology Group) score, history of smoking, history of COPD, history of diabetes mellitus, pathological diagnosis, Ki67, tumor site, TMN stage, whether surgery was performed, whether chemotherapy was performed, and progression. Radiologic data included enhanced lung computed tomography (CT) within 6 months after the completion of RT and evaluation of radiologic characteristics of SARP within half a year after radiotherapy.

### Radiation therapy and physical dosimetric factors

All lung cancer patients received three-dimensional conformal RT (3D-CRT) or intensity-modulated radiation therapy (IMRT). All radiotherapy plans were generated by a therapist on Monaco (Elekta, version 5.11.03) or Eclipse (Varian Medical Systems, version 13.6) treatment planning systems. The X-ray beam energy was 6 MV. The requirements were as follows: the prescribed dose is achieved in at least 95% of the PTV; ≥110% dose hot spots are absent outside of the PTV. The dose constraints were defined for organs at risk as follows: Mean lung dose(MLD) < 20 Gy; total lungs: V5 < 60%, V20 < 30%, V30 < 20%; Heart: V30 < 40%, V40 < 30%; Maximum point dose of spinal cord < 45 Gy. The delineation of target volumes and organs at risk was in accordance with the Radiation Therapy and Oncology Group guidelines [14]. Physical dosimetric parameters were dose volume histogram (DVH) parameters extracted from the treatment planning systems. These included V5, V10, V15, V20, and V30 of the ipsilateral lung, contralateral lung, and total lung, mean total lung dose (Dmean), mean heart dose, maximum spinal cord dose, and the ratio between the planning target volume (PTV), and total lung volume (LV).

### Chemotherapy

All lung cancer patients received personalized treatment strategy of synchronous or sequential chemotherapy based on the pathological diagnosis. The chemotherapy regimens included cisplatin combined with etoposide, pemetrexed combined with carboplatin, or cisplatin combined with paclitaxel. These regimens are widely used in clinical practice [15, 16]. Dosing was performed once every 21 days for the chemotherapy regimens, and the median cycle was 3 weeks (range: 2–4 weeks). All doses and modifications of the chemotherapy regimen were performed in accordance with the Chinese Society of Clinical Oncology and NCCN guidelines.

### RP evaluation

We retrospectively analyzed the clinical data and radiologic examinations of all lung cancer patients before and after radiotherapy. Most patients underwent one chest CT follow-up every 3 months. The diagnosis of RP was mainly confirmed through clinical symptoms, physical examination, and chest radiologic examinations. Pneumonitis was defined as follows: (1) localized pneumonitis in which the timing and symptoms were consistent with radiotherapy and required the use or extension of steroid treatment; (2) radiologic presentation of pneumonitis consistent with the Irradiation volume. RP was graded in accordance with RTOG and/or Common Terminology Criteria for Adverse Events Version 5.0 (CTCAE v5.0) [17]. SARP was defined as grade ≥ 3 RP that occurred with 3–6 months after radiotherapy.

### Statistical analysis

Student’s t-test was used for intergroup comparisons, while a Mann–Whitney *U* test was used for data with skewed distribution. Qualitative data were presented as frequencies (%), and Fisher’s exact test or *χ*^2^ test was used for intergroup comparison of nonparametric data. A Mantel–Haenszel *χ*^2^ and Mann–Whitney *U* test were used for intergroup comparison of ordinal data. A binary logistic regression model was used for univariate and multivariate analysis to evaluate the relationship between SARP patient outcomes (death or survival) and clinical factors and physical dosimetric characteristics. The hazard ratio, 95% confidence interval, and P value were reported. All data were computed using the Statistical Package for Social Science program (SPSS for Windows, version 20.0, SPSS Inc, Chicago, IL, USA) and R software (version 3.5.3). P < 0.05 was considered statistically significant.

## Results

### Clinical characteristics of deceased and surviving SARP patients

In this study, the clinical characteristics of lung cancer radiotherapy patients who developed SARP were retrospectively analyzed. Table 1 describes the patient, disease, and treatment characteristics. No significant differences were observed between the death cohort and the survivor cohort (all P > 0.05). In the included 66 SARP patients, the median age was 64.50 years. Most of the patients (n = 52, 78.79%) had stage III disease, while 14 (21.21%) patients had stage II disease at diagnosis and continued to receive postoperative adjuvant radiochemotherapy. All these patients underwent chemotherapy, and 20 (30.30%) of them underwent surgery. All patients received 40–65 Gy lung and/or mediastinal radiotherapy. Among them, 24 patients received 40-58 Gy/20-28f segmented radiotherapy, 33 patients received 59-60 Gy/30f segmented radiotherapy, and 9 patients received 61-65 Gy/32f segmented radiotherapy.

**Table 1:** Clinical characteristics of SARP patients

Clinical characteristic	Total (N = 66)	Deceased patients (n = 31)	Surviving patients (n = 35)	P value
Age (years)				
Median (IQR)	64.50 (63.00–67.00)	66.00 (62.00–71.00)	64.00 (62.00–67.00)	0.175
Gender				0.263
Male	49.00 (74.24%)	25.00 (80.65%)	24.00 (68.57%)	
Female	17.00 (25.76%)	6.00 (19.35%)	11.00 (31.43%)	
ECOG PS				0.948
0–1	53.00 (80.30%)	25.00 (80.65%)	28.00 (80.00%)	
2	13.00 (19.70%)	6.00 (19.35%)	7.00 (20.00%)	
COPD				0.896
Yes	25.00 (37.88%)	12.00 (38.71%)	13.00 (37.14%)	
No	41.00 (62.12%)	19.00 (61.29%)	22.00 (62.86%)	
Smoking (pack-year)				0.828
< 20	35.00 (53.03%)	16.00 (51.61%)	19.00 (54.29%)	
≥ 20	31.00 (46.97%)	15.00 (48.39%)	16.00 (45.71%)	
Diabetes				0.855
Yes	8.00 (12.12%)	4.00 (12.90%)	4.00 (11.43%)	
No	58.00 (87.88%)	27.00 (87.10%)	31.00 (88.57%)	
Histology				
SCC	38.00 (57.58%)	18.00 (58.06%)	20.00 (57.14%)	0.997
AC	13.00 (19.70%)	6.00 (19.35%)	7.00 (20.00%)	
Other	15.00 (22.72%)	7.00 (22.58%)	8.00 (22.86%)	
Ki-67				0.367
Median (IQR)	60.00 (40.00–60.00)	40.00 (0.00–60.00)	65.00 (40.00–80.00)	
Lobe				
Right middle	8.00 (12.12%)	2.00 (6.45%)	6.00 (17.14%)	0.342
Right upper	22.00 (33.33%)	10.00 (32.26%)	12.00 (34.29%)	0.862
Right lower	16.00 (24.24%)	9.00 (29.03%)	6.00 (17.14%)	0.250
Left upper	15.00 (22.72%)	7.00 (22.58%)	8.00 (22.86%)	0.979
Left lower	6.00 (9.09%)	3.00 (9.68%)	3.00 (8.57%)	1.000
AJCC 8 Stage				0.728
II	14.00 (21.21%)	6.00 (19.35%)	8.00 (22.86%)	
III	52.00 (78.79%)	25.00 (80.65%)	27.00 (77.14%)	
Prior lung cancer surgery				0.454
Yes	20.00 (30.30%)	8.00 (25.81%)	12.00 (34.29%)	
No	46.000 (69.70%)	23.00 (74.19%)	23.00 (65.71%)	
Chemotherapy				0.767
Concurrent chemotherapy	50.00 (75.76%)	24.00 (77.41%)	26.00 (74.29%)	
Sequential chemotherapy	16.00 (24.24%)	7.00 (22.58%)	9.00 (25.71%)	
RT dose received				0.127
40 or 58 Gy	24.00 (36.36%)	10.00 (32.26%)	14.00 (40.00%)	
59–60 Gy	33.00 (50.00%)	19.00 (61.29%)	14.00 (40.00%)	
61–65 Gy	9.00 (13.64%)	2.00 (6.45%)	7.00 (20.00%)	

### Physical dosimetric characteristics of SARP patients

SARP had developed in 31 patients who died and in 35 surviving patients. To further analyze the differences between deceased patients and surviving patients with SARP, we conducted a statistical analysis of physical dosimetric parameters. Table 2 describes the physical dosimetric parameters of the patients. By analyzing the physical dosimetric parameters of deceased and surviving patients, we found that there were statistically significant differences in ipsilateral lung V5 and V30, contralateral lung V5, V10, and V30, total lung V5, V10, and V30, mean lung dose, mean heart dose, and maximum spinal cord dose between the two groups (all P < 0.05).

**Table 2:** Physical dosimetric characteristics of SARP patients

Dose parameter characteristic	Total (N = 66)	Deceased patients (n = 31)	Surviving patients (n = 35)	P value
Ipsilateral lung				
V_5_ (%)	48.26 (39.58–53.93)	51.23 (43.02–58.31)	39.58 (16.35–53.90)	0.012
V_10_ (%)	38.21 (30.72–44.22)	41.37 (34.56–50.51)	30.72 (21.26–44.90)	0.064
V_15_ (%)	33.36 (31.04–39.12)	32.64 (23.84–40.40)	36.05 (31.54–41.00)	0.412
V_20_ (%)	30.81 (27.33–33.74)	32.34 (26.50–36.50)	29.27 (26.69–33.74)	0.507
V_30_ (%)	22.64 (18.70–27.42)	28.30 (23.41–33.58)	20.36 (16.56–22.53)	0.009
Contralateral lung				
V_5_ (%)	24.89 (18.48–30.27)	28.31 (23.98–36.47)	15.06 (5.73–30.82)	0.002
V_10_ (%)	16.04 (12.03–20.30)	17.97 (14.10–22.72)	14.19 (3.50–21.71)	0.031
V_15_ (%)	12.01 (8.71–15.70)	11.49 (2.86–17.11)	14.13 (8.79–17.23)	0.188
V_20_ (%)	8.78 (6.24–11.48)	7.92 (4.60–11.05)	9.82 (6.43–13.87)	0.926
V_30_ (%)	4.48 (2.96–7.66)	7.56 (2.94–15.65)	3.54 (2.25–7.18)	0.025
Total lung				
V_5_ (%)	32.74 (26.94–39.08)	39.04 (31.62–43.80)	16.77 (8.741–35.70)	0.000
V_10_ (%)	24.42 (21.06–29.18)	29.18 (23.06–32.78)	20.88 (12.74–25.06)	0.004
V_15_ (%)	21.49 (18.15–25.78)	19.06 (15.35–26.60)	24.38 (18.42–27.74)	0.182
V_20_ (%)	19.39 (16.19–21.81)	19.28 (14.21–25.80)	20.00 (14.90–21.81)	0.276
V_30_ (%)	13.79 (11.67–16.58)	22.95 (15.61–28.90)	12.14 (9.29–13.38)	0.001
Mean total lung dose (cGy)	978.80 (728.80–1116.00)	1069.00 (947.50 − 1218.00)	713.40 (433.60–1113.00)	0.010
Mean heart dose (cGy)	690.70 (487.00–909.70)	855.50 (428.50–1776.00)	537.10 (384.50–866.70)	0.014
Heart volume (cm^3^)	589.60 (556.30–656.10)	567.70 (517.90–630.80)	629.30 (567.60–731.70)	0.164
Maximum spinal cord dose (cGy)	2021.00 (1494.00–2376.00)	2781.00 (2015.00–4095.00)	1526.00 (1363.00–2026.00)	0.003
PTV/LV	0.18 (0.17–0.21)	0.18 (0.15–0.21)	0.19 (0.17–0.23)	0.865
Segmentation model (routine segmentation/large segmentation)	52/14	24/7	28/7	0.163

### Univariate and multivariate analysis of factors that affect the prognosis of SARP patients

The clinical factors and physical dosimetric parameters of deceased and surviving SARP patients were considered for univariate and multivariate analyses. Table 3 summarizes the predictor variables of outcomes in SARP patients in univariate analysis. The potential predictors of SARP death were as follows: ipsilateral lung V5 and V30, contralateral lung V5, V10, and V30, total lung V5, V10, and V30, MLD, mean heart dose, and maximum spinal cord dose. Multivariate analysis (Table 4) showed that ipsilateral lung V5 (OR, 0.225; 95% confidence interval: 0.051–0.993; P < 0.05) and total lung V5 (OR, 40.976; 95% confidence interval: 1.029–1632.127; P < 0.05) were the predictors of the final outcome of SARP patients.

**Table 3:** Univariate analysis of clinical and physical dosimetric factors predicting the clinical outcomes of SARP

Parameter	P value	OR	95% CI
Clinical characteristics			
Age (≤ 60 vs. > 60; years)	0.267	0.524	0.167–1.640
Gender (Male vs. Female)	0.274	3.643	0.359–36.986
ECOG PS (0–1 vs. 2)	0.068	3.243	0.867–3.653
COPD (Yes vs. No)	0.432	2.238	0.179–4.226
Smoking (< 20 vs. ≥ 20; pack year)	0.630	1.038	0.461–1.129
Diabetes (Yes vs. No)	0.095	0.153	0.017–1.390
Histology			
SCC	0.997	Ref.	
AC	0.963	1.029	0.331–3.407
Other	0.978	0.980	0.221–4.343
Ki-67 (≤ 20 vs. > 20; %)	0.477	1.422	0.538–3.758
Lobe			
Left upper	0.736	Ref.	
Left lower	0.594	0.681	0.165–2.804
Right middle	0.793	0.778	0.119–5.100
Right upper	0.159	0.259	0.040–1.700
Right lower	0.603	0.707	0.191–2.613
TNM stage (II vs. III)	0.729	0.729	0.246–2.663
Prior lung cancer surgery (Yes vs. No)	0.456	1.500	0.517–4.352
Chemotherapy (concurrent vs. sequential)	0.159	5.037	0.532–47.733
RT dose received			
40 or 58 Gy	0.156	Ref.	
59–60 Gy	0.310	2.500	0.426–14.657
61–65 Gy	0.075	4.750	0.854–26.432
Ipsilateral lung			
V_5_ (%)	0.014	0.972	0.951–0.994
V_10_ (%)	0.099	0.977	0.951–1.004
V_15_ (%)	0.407	0.987	0.956–1.018
V_20_ (%)	0.501	1.011	0.978–1.046
V_30_ (%)	0.014	1.046	1.009–1.084
Contralateral lung			
V_5_ (%)	0.004	0.951	0.919–0.984
V_10_ (%)	0.037	0.953	0.911–0.997
V_15_ (%)	0.189	0.967	0.920–1.017
V_20_ (%)	0.925	0.998	0.950–1.047
V_30_ (%)	0.034	1.053	1.004–1.105
Total lung			
V_5_ (%)	0.001	0.939	0.906–0.974
V_10_ (%)	0.006	0.931	0.884–0.98
V_15_ (%)	0.181	0.961	0906–1.019
V_20_ (%)	0.261	1.033	0.976–1.093
V_30_ (%)	0.003	1.104	1.035–1.178
MLD (cGy)	0.012	0.998	0.997–1.000
Heart mean dose (cGy)	0.023	1.001	1.000–1.001
Heart volume (cm^3^)	0.176	0.998	0.996–1.001
Maximum spinal cord dose (cGy)	0.004	1.001	1.000–1.001
PTV/LV	0.862	0.714	0.016–32.166
Segmentation model (routine segmentation/large segmentation)	0.798	1.167	0.358–3.801

**Table 4:** Multivariate analysis of predictors of clinical outcomes of SARP

Parameters	P value	OR	95% CI
Ipsilateral lung			
V_5_ (%)	0.049	0.225	0.051–0.993
V_10_ (%)	0.236	8.961	0.239–335.900
V_30_ (%)	0.343	0.395	0.058–2.692
Contralateral lung			
V_5_ (%)	0.057	0.152	0.022–1.058
V_10_ (%)	0.268	14.482	0.128–1642.168
V_30_ (%)	0.904	1.153	0.115–11.549
Total lung			
V_5_ (%)	0.048	40.976	1.029–1632.127
V_10_ (%)	0.209	0.003	0.000–25.700
V_30_ (%)	0.286	9.601	0.151–610.532
Mean total lung dose (cGy)	0.121	0.986	0.969–1.004
Mean heart dose (cGy)	0.136	1.004	0.999–1.008
maximum spinal cord dose (cGy)	0.705	1.000	0.999–1.002

### Analysis of intergroup differences in time of SARP onset between deceased and surviving patients

**Fig. 1:**
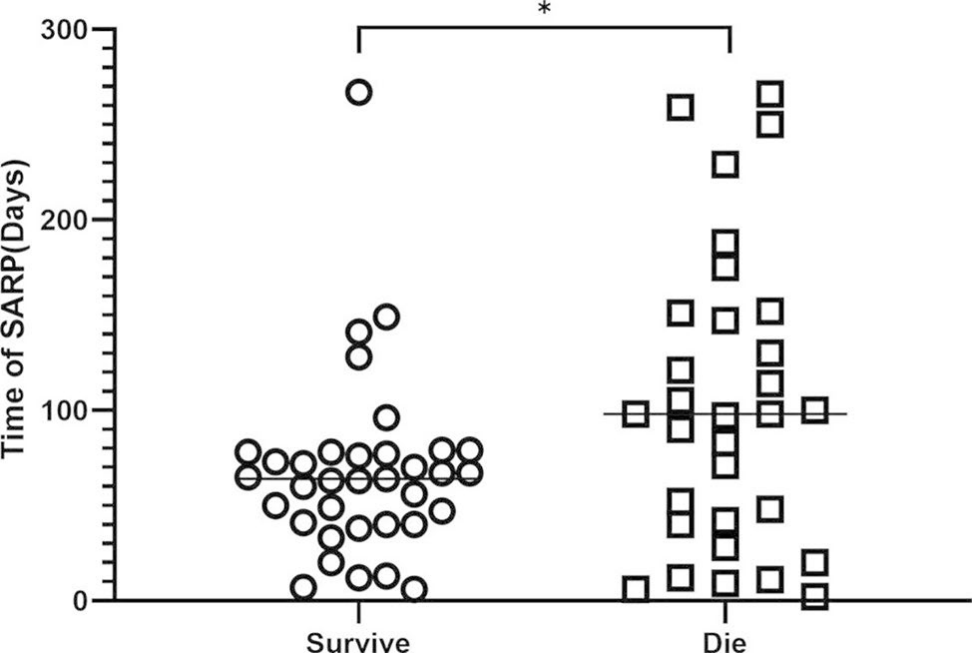
The difference of SARP time between the two groups was analyzed(*p = 0.033)

To further analyze whether there are differences in the time of SARP onset between deceased and surviving patients, we compared the time from RT completion to SARP onset between the two groups. We found that the P value was 0.033, which was statistically significant (Fig. 1). The median time to SARP onset was 64 days (95% CI: 49–73) in the survival group and 98 days (95% CI: 48–130) in the death group. The median time from SARP onset to death was 159.50days (95% CI: 100–266) in the death group, and the median time from SARP onset to outcome was 93.50 days (95% CI: 60–117) in the survival group.

## Discussion

Radiation pneumonitis(RP) is a common and potentially fatal dose-limiting toxicity that occurs in lung cancer patients undergoing lung and/or mediastinal radiotherapy or synchronous radiochemotherapy, and its incidence is 14.6–37.2%. Most cases occur around 6 months after radiotherapy [18, 19]. RTOG classified RP into grades 0 to 5 based on the clinical symptoms, lung radiologic presentation, and corticosteroid treatment results. Grade 3 and above RP is classified as severe acute RP(SARP) [8, 17]. With advances in radiotherapy, the indications for chest radiotherapy in lung cancer patients are currently expanding, and the incidence of radiotherapy-induced severe lung complications has decreased. However, SARP still occurs after lung cancer radiotherapy with an incidence rate of 3–6% [18, 20]. In clinical practice, the clinical outcomes of such patients were different even though they were promptly discovered and aggressive clinical treatment was carried out. Some patients recovered, while symptoms gradually worsened in some patients and they died. There have been no related studies that compared the radiation dosimetric parameters between lung cancer patients who developed SARP with different outcomes and evaluated their role in treatment-related toxicity. This study was the first to reveal the heterogeneity in clinical factors, physical dosimetric factors, and time of SARP onset in SARP patients. This could provide a theoretical research foundation for further optimization of lung radiotherapy plan and decrease the incidence of fatal RP in lung cancer patients in clinical practice.

In this study, the data of 31 patients who had undergone lung and/or mediastinal radiotherapy and died of SARP from January 2013 to December 2019 were collected. In addition, data were collected from 35 patients who developed SARP and survived during the same period. It is worth noting that we tried as much as possible to collect patients who had undergone chest and/or mediastinal radiotherapy and died of SARP. However, we did not include all patients who developed SARP and survived because equal sampling was employed in this study and the surviving patients that were included to the analysis could represent the overall characteristics of all patients who developed SARP and survived. Therefore, the number of patients in this analysis does not represent the mortality rate of SARP in our center. In the existing studies on lung cancer patients who develop RP after radiotherapy, some clinical characteristics have been considered important risk factors for RP progression. Previous studies have shown that performance status, history of smoking, nutritional status, past lung disease, tumor stage, pathology and site, concurrent chemotherapy, pre-radiotherapy lung function, and surgery are associated with the occurrence and severity of RP [7, 21–27]. According to Giuranno et al., older age (> 65 years) is a factor for decreased tolerability to radiotherapy [17, 28], and women may have a higher risk of RP due to their lower lung volume [29]. A retrospective study in a cancer center in China showed that the incidence of fatal RP was higher in lung cancer patients with chronic silicosis. In lung cancer patients, symptomatic interstitial lung disease (ILD), even asymptomatic subclinical ILD, is a risk factor for RP [30, 31]. There is still debate over the roles of COPD and lung infection. A prospective study by Zhou et al. showed that emphysema was a risk factor for RP after radiotherapy in non-Small Cell Lung Cancer patients [32], but this correlation was not found in other studies [33]. A large number of studies evaluated smoking. Surprisingly, the risk of RP in smokers is decreased. This may be due to decreased inflammatory responses, which do not respond to DNA damage [19, 28, 31]. The risk of RP in patients whose radiotherapy target region is the lung field is three times higher than that in patients whose radiotherapy target region is the mediastinum [18, 21]. At present, there are related papers on clinical factor prediction model for SARP [14, 34, 35]. In this study, we did not observe significant differences in these clinical factors between the death and the survivor cohorts (all P > 0.05). In future studies, the sample size should be expanded, and more well-designed prospective randomized controlled studies should be carried out to further analyze and validate our conclusions.

It has been widely observed and reported that DVH dose parameters are associated with RP. Multiple doses, including mean lung dose and the percentage volume that received a fixed dose, are associated with an increased risk of SARP. Previous studies have found that MLD shows a significant positive correlation with RP [36]. Hernando found that the risk of RP in patients who received an MLD of more than 8.5 Gy was 3.8 times higher than that in patients who received ≤ 8.5 Gy [37]. In our SARP patients, the MLD of the death group and the survivor group was 10.69 Gy (9.48–12.18 Gy) and 7.13 Gy (4.34–11.13 Gy), respectively, and the mean MLD of the death group was slightly higher than that of the survivor group. This result is consistent with previous data. It is interesting to observe significant differences in MLD between the two groups (P < 0.01). Univariate analysis showed that OR of MLD was 0.998 (95% CI: 0.997–1.000; P < 0.05), which means that MLD can be used as a potential evaluation marker for the final outcome of SARP patients. However, MLD did not reach significance in multivariate analysis, showing that it may not play a critical role in SARP outcome. Therefore, we will carry out more prospective studies in the future for validation. We will also confirm the optimal cutoff point for dosimetric standard to more accurately predict the final outcome of SARP patients.

The lungs are “parallel organs” as they consist of multiple parallel functional units. Other functional units are not affected when one functional unit is damaged [38]. Therefore, some studies have found that the severity of RP is intimately associated with the lung volume that received a radiation dose that exceeded the radiation tolerability of the lungs. The specific marker reflecting this relationship is PTV/LV [26, 39]. Many previous studies have shown that PTV/LV plays a critical role in RP progression. According to Jinming Yu, et al., PTV/LV is independent from other dosimetric factors and is considered a novel and special marker for SARP after radiotherapy in esophageal cancer patients [34]. In our study, no significant difference in the PTV/LV ratio was observed between the SARP death and survivor cohorts. Univariate analysis showed that OR for PTV/LV was 0.714 (95% CI: 0.358–3.801; P = 0.862), meaning that PTV/LV cannot predict the final outcome of SARP patients.

Many studies have shown that the mean V20 and V30 of patients with RP were 42.0% and 38.0%, respectively, which were higher compared with patients who did not develop RP [40]. Currently, there is widespread acceptance of V20 evaluation of treatment plans and prediction of RP incidence [41]. Hanania AN et al. reported that V20 was not only associated with the incidence of RP but also significantly correlated with the severity of RP [17]. Some researchers have found that the V20 of patients who died of RP was ≥ 32%, and the V20 of patients who developed grade 3 and above RP was ≥ 30%. V20 should be less than 25% in order to prevent SARP [18, 42, 43]. Our results showed that the V20 of ipsilateral lungs in deceased SARP patients was 32.34 (26.50–36.50), and the V20 of ipsilateral lungs in surviving patients was 29.27 (26.69–33.74), which revalidates the above findings. However, no significant differences in V20 were observed in the ipsilateral lung, contralateral lung, or total lung between these two cohorts. Hence, V20 is not a decisive factor for the final outcome of SARP patients. The study by Marks LB et al. showed that the incidence of RP was 6% and 24% when lung V30 was 17.70% and 17.80–24.50%, respectively. Therefore, the lung V30 should be controlled at below 18% [18, 43, 44]. In our study, the mean ipsilateral lung V30 of the two groups was greater than 18%; specifically, the ipsilateral lung V30 of the death group and the survivor group was 28.30 (23.41–33.58) and 20.36 (16.56–22.53), respectively. The univariate analysis of V30 in these two groups showed significant differences, but multivariate analysis did not confirm the role of ipsilateral lung V30 in the final outcome of SARP patients. Although significant differences in contralateral lung and total lung V10, mean heart dose, and maximum spinal cord dose were found in univariate analysis, they were not significant in multivariate analysis.

With advances and developments in the IMRT era, researchers have paid more attention to the effect of low dose regions in the lungs on the development of radiation pneumonia, particularly V5 [45]. Many researchers have found that limiting V5 to 60% and below can greatly decrease the incidence of RP and radiation fibrosis in patients [46]. However, some studies on the occurrence and progression of RP have not observed significant differences in V5. The multivariate analysis reported by Lu Wang et al. showed that V5 did not play an important role in SARP progression. Our study showed that the ipsilateral lung, contralateral lung, and total lung V5 were all below 60%, and there was a significant difference between the death and the survivor groups in univariate analysis. Surprisingly, multivariate analysis showed significant differences in ipsilateral lung V5 (OR, 0.225; 95% CI: 0.051–0.993; P < 0.05) and total lung V5 (OR, 40.976; 95% CI: 1.029–1632.127; P < 0.05). Finally, we believe that these two factors are independent predictors and reliable parameters of the outcome of SARP patients and jointly determine the final outcome of SARP patients. Higher ipsilateral lung and total lung V5 in the DVH are associated with greater susceptibility to SARP and poorer final outcome or death. However, we hope to increase our sample size and conduct prospective studies to decrease selection bias and further increase the reliability of the data.

Our study showed that the median time to SARP onset in the survivor group and the death group was 64 days and 98 days, respectively. The difference in the time of SARP onset between the two groups was statistically significant, and the median time of SARP onset in the survivor cohort was significantly earlier than that in the death cohort. This may be due to the pathophysiological developmental trends of RP, and patients with earlier SARP onset have significant treatment advantages as they receive treatment earlier. Therefore, these patients have better outcomes. We also found that the median time from SARP onset to death was 159.5 days in the death group, and the median time from SARP onset to outcome was 93.5 days in the survivor group. Although this study was the first to reveal the heterogeneity in clinical factors, physical dosimetric factors, and time of SARP onset in SARP patients, there are several limitations that need to be urgently solved. First, the sample size of this study was small, and the sample size should be expanded in future studies to further validate our prediction results. Furthermore, this was a retrospective study, and selection bias may be present. Lastly, we only analyzed basic clinical factors and physical parameters. In subsequent studies, bioinformatics, genome information, and radiomics information will be added to improve the prognosis determination criteria for SARP patients.

## Conclusions

In this study, there was no decisive correlation between clinical characteristics and SARP outcome (i.e., death or survival) in lung cancer patients who had undergone radiotherapy. Physical dosimetric factors, such as ipsilateral lung V5 and V30, contralateral lung V5, V10, and V30, total lung V5, V10, and V30, MLD, mean heart dose, and maximum spinal cord dose may be potential predictors of death in SARP. Multivariate analysis showed that ipsilateral lung V5 and total lung V5 were independent predictors of death in SARP patients. These ultimately determine the outcome of patients after SARP occurrence, i.e., death or survival.the median time of SARP onset in the survivor cohort was earlier than that in the death cohort. In summary, this study is the first to provide a theoretical research foundation for further optimization of chest radiotherapy plan and decreasing the incidence of fatal RP in lung cancer patients.

## Data Availability

The datasets used and/or analysed during the current study are available from the corresponding author on reasonable request.
